# Support the Journals

**Published:** 1888-06

**Authors:** 


### SUPPORT THE JOURNALS.

In the report of the meeting of the Illinois State Dental Society, in this number, will be found some sensible and timely remarks concerning the duty that dentists owe to their professional journals. There can be no "profession" without a literature of its own; the very term implies literary culture. If those engaged in any vocation are doing scientific work, there must be journals of some kind to make a permanent record of what is done, and those journals will usually be a faithful index of the status of those whom they represent. The stream cannot rise above its fountain head, and if dentists desire a literature that will be a credit to them in the eyes of the world, that shall produce the impression that they are thinking, studious men, the journals must be supported, not only with subscriptions, but by contributions from the writers of the profession. Our dental journals usually have a large exchange list of medical journals, and the impression that is made upon the editors of those journals, upon the representative men in medicine, depends to a large extent upon the manner in which they see our professional literature sustained. If any journal that stands as a representative of the profession to which it belongs shall mainly be made up of extracts from other journals, and evinces a dearth of original thought and a lack of original communications from those who should be its contributors, it marks a low tide of professional interest and reflects severely upon professional status.

